# Multiscale models driving hypothesis and theory-based research in microbial ecology

**DOI:** 10.1098/rsfs.2023.0008

**Published:** 2023-06-09

**Authors:** Eloi Martinez-Rabert, William T. Sloan, Rebeca Gonzalez-Cabaleiro

**Affiliations:** ^1^ James Watt School of Engineering, Infrastructure and Environment Research Division, University of Glasgow, Advanced Research Centre, Glasgow, UK; ^2^ Department of Biotechnology, Delft University of Technology, Delft, The Netherlands

**Keywords:** mathematical modelling, *in-silico* bottom-up methodology, microbial communities, microbial ecology

## Abstract

Hypothesis and theory-based studies in microbial ecology have been neglected in favour of those that are descriptive and aim for data-gathering of uncultured microbial species. This tendency limits our capacity to create new mechanistic explanations of microbial community dynamics, hampering the improvement of current environmental biotechnologies. We propose that a multiscale modelling bottom-up approach (piecing together sub-systems to give rise to more complex systems) can be used as a framework to generate mechanistic hypotheses and theories (*in-silico* bottom-up methodology). To accomplish this, formal comprehension of the mathematical model design is required together with a systematic procedure for the application of the *in-silico* bottom-up methodology. Ruling out the belief that experimentation before modelling is indispensable, we propose that mathematical modelling can be used as a tool to direct experimentation by validating theoretical principles of microbial ecology. Our goal is to develop methodologies that effectively integrate experimentation and modelling efforts to achieve superior levels of predictive capacity.

## 1. Introduction

Hypothesis testing as a scientific approach in environmental microbiology and biotechnology is bounded by the intrinsic complexity of microbial communities. Theory-based research is relegated by an increasing number of microbial ecology studies that focus on descriptive experiments of uncultured microbial species. However, critically testing ecological hypotheses requires rigorous experimental design while the application of novel molecular technologies for data collection has led to a multitude of top-down research approaches where data are just described [1]. Generation of knowledge through induction (e.g. accumulative characterization of uncultured microbial species) does not *per se* translate in new theoretical/mechanistic explanations for community assembly or specific fitness traits.

We propose the development of research focused on microbial ecology quantification, which driven by theoretical hypotheses, is further validated by interplay within mathematical modelling and laboratory experimentation. We describe a modelling methodology based on a bottom-up approach (piecing together sub-systems to give rise to more complex systems) in order to generate, together with experimental validation, new hypotheses and theories. By using theoretical platforms, we can target the minimization of complexity associated with natural communities directing research exploration in a more efficient way. To understand the implications associated with this methodology, we first discuss the actual position of mathematical models and experimentation in scientific research.

## 2. Experimental and theoretical models

Considering that any mathematical model represents a conceptualization of reality, it is commonly assumed that experiments should precede any modelling exercise. Modelling is then mostly placed as an alternative complement to experimentation because theoretical results must be demonstrated or validated. Nevertheless, experimental outcomes must also be demonstrated by replication and reproducibility as a major principle underpinning the scientific method. The results obtained by an experiment, an observational study, or in a statistical analysis of a dataset can be considered reliable only if these studies are replicated [2].

**Figure 1.**
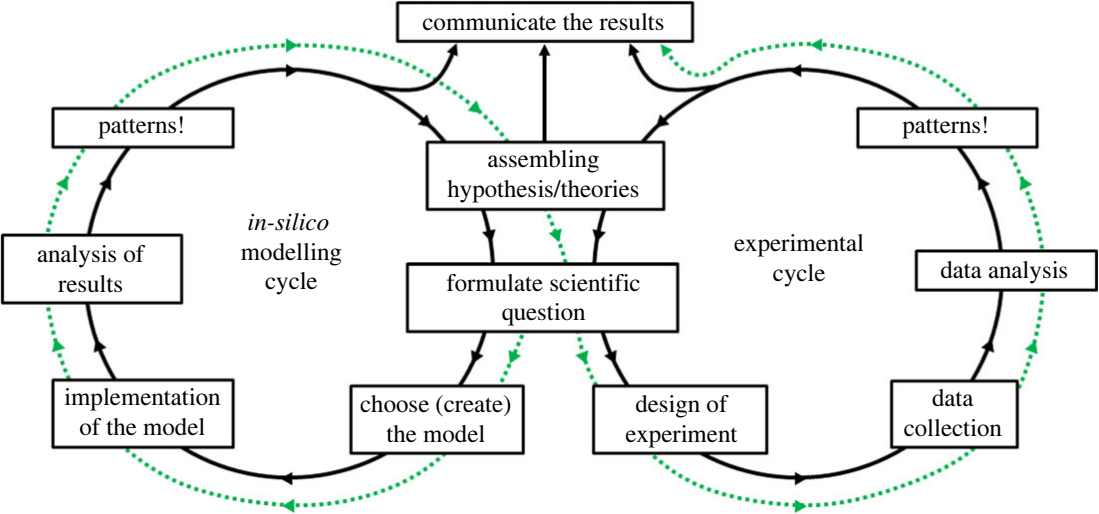
Modelling–experimental cycle. Integrated development of experimental and modelling methodologies can lead to higher levels of predictive capacities and operation control. Dotted arrow depicts the methodology presented here—theoretical model before experimentation.

Experimentation and modelling exercises might not be seen as exclusive, but interconnected methodologies (figure 1). A modelling exercise can help in defining experimental designs that validate hypotheses theoretically constructed (dotted arrow, figure 1). This level of definition also aids reproducibility, especially when applied to complex systems. It can be argued that the most useful models are constructed on the basis of the theoretical knowledge we possess [3,4], directing experimentation that aims at validating the principles on which they are built, that is, using mathematical models as hypotheses generator.

## 3. *In-silico* bottom-up methodology

When modelling continuous and complex natural processes, they can be treated as a group of discrete elements interconnected able to define observable events that can be measured. A bottom-up approach is essentially piecing together sub-systems to give rise to more complex systems. *In-silico* models that follow a bottom-up approach aim to explain how emerging properties of complex communities arise from simpler processes [5].

**Figure 2.**
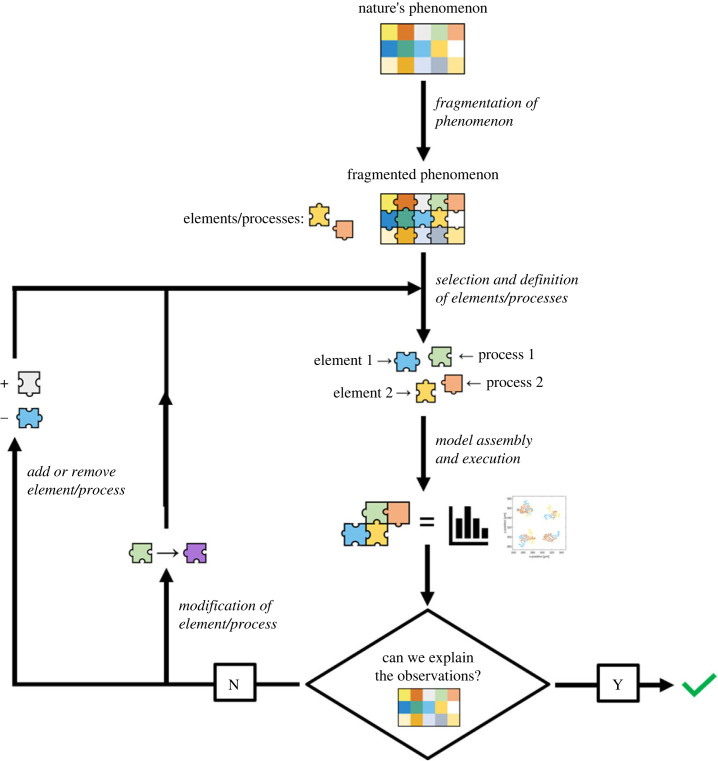
Schematic of *in-silico* bottom-up methodology.

The first step to build an *in-silico* bottom-up methodology is to identify all the elements that describe a particular phenomenon (i.e. the *fragmentation*; figure 2). After that, the elements that will be part of the model are selected. For this step having enough information is crucial, either providing enough specific experimental data or by means of construction of theories and first principles (generally associated with a set of mathematical equations). Additionally, in the process of selection of elements one must consider and evaluate the model complexity and the possibilities for experimental validation [6]. Subsequently, the mathematical model is assembled*.* A mathematical model is a conceptual representation of a mechanism (or a collection of them) limited by our knowledge about the reality. All models are constituted by the quintuple:

— *Domain* (*D*): set of factual items (elements and processes) that constitute the studied system.
— *Scientific question* (*Q*): question(s) that states the reason for modelling and the construction of the model.
— *Interpretation* (*I*): validated explanations of each item of the *domain*. Definition of spatial scale(s) and temporal extent is included here.
— *Assumptions* (*A*): set of explicitly stated (or implicit premised) conventions and choices that fulfils the holes in our *interpretation* of reality. These establish the limits of our model and simplify the problem (e.g. by ignoring some processes or elements that cannot be well described).
— *Formalism* (*F*): set of mathematical expressions that represent the items of the *domain*.

The definition of each of the components 〈*D*, *Q*, *I*, *A*, *F*〉 is fundamental for the success of the modelling process. The construction of the mathematical model starts with the abstraction of the current knowledge about the *domain* (*D*). Based on our understanding, the *scientific question* (*Q*) is stated. Then, the formalization of our knowledge about the *domain* is addressed, defining *interpretation, assumptions* and *formalism* (i.e. the modelling approach, *I*, *A*, *F*). Table 1 shows an example of the statement of 〈*D*, *Q*, *I*, *A*, *F*〉. The limits of the modelling approach 〈*I*, *A*, *F*〉 are established by the scope of the fundamental processes and the selected elements. The overlook of a key element or process can make our model inaccurate. An example of this is the omission of the diffusion in an aggregated system as presented in *Model 1* in table 1. Although an NH_3_-limiting environment was considered (this being one of the main pressure factors for the selection of Comammox process [7]), the enrichment observed in the Daims *et al.* study [8] was not predicted with *Model 1*. Therefore, a model will be *useful* (i.e. generates reliable knowledge) if and only if there is no discrepancy between the results of the modelling approach 〈*I*, *A*, *F*〉 and the observations in the real *domain* (*D*).

**Table 1.** Example of statement of model components 〈*D, Q, I, A, F*〉. Legend *μ*_max_, maximum specific growth rate; $K_{{\mathrm{NH}}_{3}}$, half-saturation constant for NH_3_; $K_{O_{2}}$, half-saturation constant for O_2_; *a_m_*, specific maintenance rate; [NH_3_], [O_2_], substrate concentration; D, diffusion coefficient; *R_xy_*, reaction term in each discretized space; *R*_BL_, reaction term in bulk liquid; HRT, hydraulic retention time; *X,* bacteria concentration.

Model 1 (ODE system)^a^	Model 2 (individual-based model)^b^
***domain** (D)*	
suspended microbial community growing under a dynamic ammonia environment simulated as a continuous stirred tank reactor (CSTR) until it reaches steady state (1825 days). Dense population of cells was generated (simulated as small aggregates)	
***scientific question/aim of modelling** (Q)*	
*in-silico* prediction of the selection of Comammox (from NH_3_ to NO_3_) over canonical ammonia oxidation (from NH_3_ to ${\mathrm{NO}}_{2}^{-}$) under NH_3_-limiting environment	
***interpretation** (I)*	
aggregates are too small and diffusion gradients for chemical components are neglected	diffusion gradients for chemical components are computed
*spatial scales*	
individual scale: biomass concentration of reactor	individual scale: independent entities
micro-scale: *not considered*	micro-scale: aggregate region + boundary layer
macro-scale: bulk liquid	macro-scale: bulk liquid
***assumptions** (A)*	
no kinetic competition—same growth kinetics both metabolic activities considered ($\mu_{\max},K_{{\mathrm{NH}}_{3}},K_{O_{2}},a_{m}$)	
different growth yield (*Y_X_*_/*S*_)—estimated according to bioenergetic analysis	
ideal behaviour of the CSTR operation	
***formalism** (F)*	
microbial growth: $\mu_{\max}=0.01\;h^{-1};\;K_{{\mathrm{NH}}_{3}}=1.0\;\mu M;\;K_{O_{2}}=3.13\;\mu M;\;a_{m}=0.001\;h^{-1}$	
growth yield (*Y_X_*_/*S*_): ammonia oxidation, 4.09 × 10^−2^ mol*_X_*/mol*_N_*; Comammox, 6.51 × 10^−2^mol*_X_*/mol*_N_*	
bacteria growth rate (Monod): $\mu =\mu_{\max}\cdot \frac{\left[{\mathrm{NH}}_{3}\right]}{K_{{\mathrm{NH}}_{3}}+[{\mathrm{NH}}_{3}]}\cdot \frac{\left[O_{2}\right]}{K_{O_{2}}+[O_{2}]}-a_{m}$	
micro-scale: *not considered*	micro-scale: second Fick's Law + reaction $\frac{\partial }{\partial t}[S](x,y)=D\cdot \nabla_{xy}^{2}[S](x,y)+R_{xy}$
macro-scale (mass balance of CSTR): $\frac{d}{dt}[S]=\frac{1}{\mathrm{HRT}}\cdot ({\left[S\right]}_{\mathrm{influent}}-[S])+R_{\mathrm{BL}};R_{\mathrm{BL}}=\left(\frac{1}{Y_{X/S}}\right)\cdot \mu (\left[S\right])\cdot X$	
**results from modelling (with triplicate)**	
ammonia oxidation: 50.0 wt%	ammonia oxidation: 14.0 wt%
Comammox: 50.0 wt%	Comammox: 86.0 wt%
**experimental results from Daims *et al.*** [8]	
after a series of sub-cultivation steps, an enrichment culture of Comammox bacteria (60–80% of the community) was obtained without known ammonia oxidizers (bacteria or archaea) in it	

^a^Model performed with MATLAB (R2020b) via the built-in function ‘ode45()’.

^b^Source code is available on public GitHub repository at https://github.com/Computational-Platform-IbM/IbM.

The outcome from the computational model is validated using the available experimental data. If the model is accurate enough to represent the system of interest, we can use it for prediction and generation of new knowledge. New theoretical knowledge can be generated by the validation of the discrete elements and processes employed. To increase the accuracy of a mathematical model, we could (i) add (or remove) elements and/or processes that were previously overlooked or (ii) modify those previously selected (iterative procedure; figure 2).

## 4. Scales of modelling for microbial communities

We define three scales that are fundamental in the modelling of microbial communities: individual scale (main elements of the model), micro-scale (processes simulated at the same resolution as individual scale) and macro-scale (elements and processes described from a larger perspective, generally embedded in the bulk liquid region). Table 1 presents an example of these scales for the modelling of microbial aggregates.

The different scales of the model are interconnected and they influence each other. For example, the microbial activity is influenced by the local conditions stated by the micro-scale and, simultaneously, the microbial cells shape the local environment. The integration of multiple scales with different characteristic times (e.g. cell division: approximately 1 h; diffusion–reaction process: approximately 10^−8^ h) is possible thanks to the use of proper time discretization and systematic resolution—a *pseudo-steady state* for processes with lower characteristic time is considered a good approximation for most applications when solving those with higher characteristic time [9]. Multiscale modelling also covers processes with a gap between characteristic space scales, such as diffusion–reaction process (approx. 10^−6^ m) and the bulk processes (approx. 10^−3^–1 m). Because the characteristic time and space are positively correlated (ensuring the numerical condition stability), the systematic resolution presented above also deals with the gap between space scales.

### 4.1. Individual scale: models that describe individual microbial activity

In microbial ecology, Monod equation has been widely used to describe biological activity [10,11]. Growth is defined by empirical parameters measured for specific populations and conditions without considering the ecological interactions or microbial evolution that would explain the specific dominant activities observed in bioprocesses.

Aware of the limitations imposed by the use of Monod equation [12], molecular systems biology attempts to comprehend cell growth through mechanistic descriptions of intracellular processes. With different levels of metabolic and physiological detail, these descriptions are able to identify some fitness trade-offs in microbial activity arising from a common set of physicochemical and intracellular constraints. Resource allocation theory defines that microorganisms optimize the use of limited intracellular resources towards expressing the most efficient strategy for growth, allowing the description of their dynamic adaptations to the environment [13].

An approach like this requires in many cases detailed physiologic and metabolic information available, generating mathematical models that require a high number of parameters. This limits their application to a few model organisms [14]. A validation of a first-principles approach can overcome the reduced empirical information by attempting the prediction of kinetic parameters for growth through mathematical equations. For example, bioenergetics analyses provide a tool for quantifying growth yields [15]. Efforts towards estimating the trends of other kinetic parameters for description of microbial activity and growth can also be considered on a framework of resource allocation [16].

### 4.2. Micro-scale: prediction of emerging properties of communities

The integration of models that describe microbial growth with the definition of the local conditions dynamically affected by the microbial activity enables the description of interactions between the media, individuals and community. This allows the prediction of emergent properties that arise from the definition of individual activity [17], and possible estimation of ecological trends in communities that can be compared to experimental observations.

Depending on the scientific question asked, abiotic physicochemical processes should be considered. Examples of this are kinetic models of acid–base reactions, chemical speciation or precipitation. The consideration of spatial competition might also be crucial to describe ecological interactions in specific communities [4].

### 4.3. Macro-scale: scaling up and down key processes

Modelling large-scale systems with micro-scale resolution (approx. 10^−6^ m) is computationally a very intensive task. To overcome this limitation, micro- and macro-scale processes are independently resolved following a systematic procedure through the establishment of *pseudo-steady states* [9]. The full integration of both spatial scales can be achieved if the micro-scale processes are scaled up, and the macro-scale processes are scaled down. The scaling-up is based on the consideration of a statistically representative volume of the larger system in full detail. It is assumed that the representative volume yields a representative influence on the whole system (i.e. the macro-scale). On the other hand, the scaling-down of macro-scale processes needs the definition of boundary conditions for the simulated system. Based on the goal that the model has (or the *scientific question* (*Q*)), the boundary conditions can be set (i) unidirectionally (only macro-scale influences micro-scale; fixed boundary conditions) or (ii) bidirectionally (macro-scale influences micro-scale and vice versa; dynamic boundary conditions).

## 5. Conclusion

An alternative avenue to advance the understanding of microbial ecology, community assembly and biological activity would aim at the deconstruction of complexity by means of a bottom-up approach, where multiscale models, robust experimental data collection, and method development are integrated. In essence, we propose the design of cultivation-based experiments that help the validation of hypotheses constructed by mathematical modelling. Although hypothesis-based cultivation experiments can be seen as too idealistic when compared with the intrinsic complexity of microbial ecology, well-designed experiments with targeted scientific questions can lead to the discovery of new metabolic characteristics or relationships between species. In this context, the integration of molecular technologies would aid the validation of theoretical hypotheses. The rationalization of ecological interactions in a community, and their relation to the environment, breaks down complexity, reduces the necessity of data, and accelerates understanding [18]. This promises a higher level of prediction capacity which can directly impact on the engineering of bioprocesses. In this effort, commonalities between communities will be found, which implies that knowledge construction in one field will benefit others (e.g. research on anaerobic digestion processes and the understanding of gut microbiome or marine microbial communities).

## Data Availability

The source code is available on the public GitHub repository at https://github.com/Computational-Platform-IbM/IbM.
